# Supplementary material for: Identification of photoperiod-induced specific miRNAs in the adrenal glands of Sunite sheep (Ovis aries)
Source: Front Vet Sci. 2022 Jul 22;9:888207. doi: 10.3389/fvets.2022.888207 (PMC9354845; doi:10.3389/fvets.2022.888207)
Supplement: Supplementary file 1 [file Data_Sheet_1.ZIP › Supplementary file/miRDeep2 file.zip!/miRDeep2 file/Know_Structure/SPLP42A1/oar-mir-125b.pdf]

miRBase precursor : oar-mir-125b
Total read count : 155269
oar-miR-125b read count : 154667
remaining reads : 602

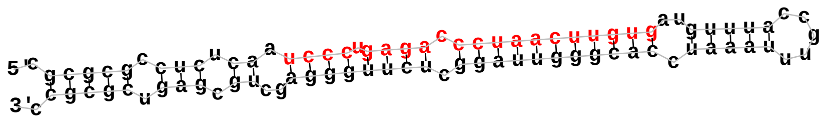

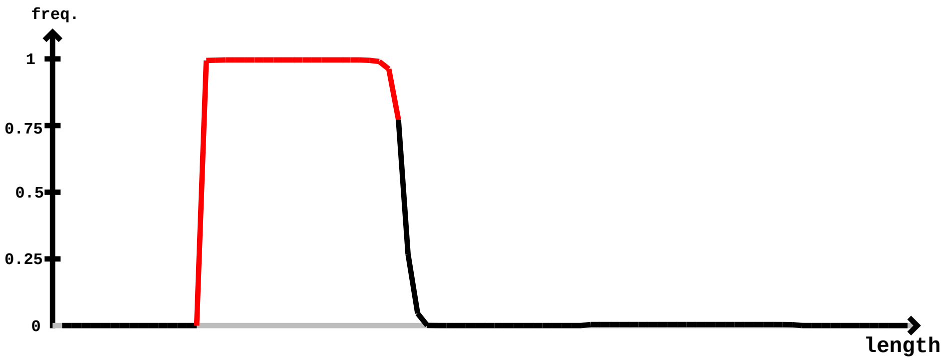


```
                     oar-miR-125b
5'- cgcgcgccucucaaucccugagacccuaacuugugauguuuaccguuuaaauccacggguuaggcucuugggagcugcgagucgcgcc   -3'    exp
    .(((((.(((.((.((((.((((.(((((((((((..(((((.....))))).))))))))))).))))))))..)).))).))))).    reads     mm       sample
    .............aucccugagacccuaacuugu......................................................    1         0        seq
    .............aucccugagacccuaacuugug.....................................................    2         0        seq
    .............Gucccugagacccuaacuuguga....................................................    1         1        seq
    ..............ucccugagacccuaCcu.........................................................    1         1        seq
    ..............ucccugagacccuaacu.........................................................    219       0        seq
    ..............ucccugagaUccuaacu.........................................................    1         1        seq
    ..............Acccugagacccuaacu.........................................................    1         1        seq
    ..............ucccugagGcccuaacu.........................................................    1         1        seq
    ..............ucccugagacccuaacC.........................................................    29        1        seq
    ..............ucccugagacccuaacCu........................................................    7         1        seq
    ..............ucccugagacccuaacuu........................................................    643       0        seq
    ..............ucccugagacccuaacuug.......................................................    4406      0        seq
    ..............ucccugagacccuaacuugu......................................................    29377     0        seq
    ..............ucccugagacccuaacuugug.....................................................    78181     0        seq
    ..............ucccugagacccuaacuuguga....................................................    34430     0        seq
    ..............ucccugagacccuaacuugugau...................................................    4103      0        seq
    ..............ucccugagacccuaacuugugaG...................................................    2868      1        seq
    ..............ucccugagacccuaacuugugaGg..................................................    30        1        seq
    ..............ucccugagacccuaacuugugaug..................................................    25        0        seq
    ..............ucccugagacccuaacuugugaugu.................................................    21        0        seq
    ..............ucccugagacccuaacuugugauguu................................................    1         0        seq
    ...............cccugagacccuaacuug.......................................................    2         0        seq
    ...............cccugagacccuaacuugu......................................................    11        0        seq
    ...............cccugagacccuaacuugug.....................................................    11        0        seq
    ...............cccugagacccuaacuuguga....................................................    121       0        seq
    ...............cccugagacccuaacuugugau...................................................    3         0        seq
    ...............cccugagacccuaacuugugaG...................................................    5         1        seq
    ................ccugagacccuaacuug.......................................................    5         0        seq
    ................ccugagacccuaacuugu......................................................    34        0        seq
    ................ccugagacccuaacuugug.....................................................    76        0        seq
    ................ccugagacccuaacuuguga....................................................    43        0        seq
    ................ccugagacccuaacuugugaG...................................................    2         1        seq
    ................ccugagacccuaacuugugau...................................................    2         0        seq
    .................cugagacccuaacuugu......................................................    1         0        seq
    .................cugagacccuaacuugug.....................................................    1         0        seq
```

oar-miR-125b

```
cgcgcgccucucaaucccugagacccuaacuugugauguuuaccguuuaaauccacggguuaggcucuugggagcugcgagucgcgcc
.................cugagacccuaacuuguga....................................................    1    0    seq
..................ugagacccuaacuugug.....................................................    1    0    seq
.....................................................cacggguuaggcucuugggag..............    1    0    seq
.....................................................cacggguuaggcucuugggagcu............    1    0    seq
......................................................acggguuaggcucuuggga...............    1    0    seq
......................................................acggguuaggcucuugggag..............    6    0    seq
......................................................acggguuaggcucuugggagc.............    61   0    seq
......................................................acggguuaggcucuugggagcu............    518  0    seq
......................................................acggguuaggcucuugggagcug...........    14   0    seq
```
